# Supplementary material for: OpenMM 7: Rapid development of high performance algorithms for molecular dynamics
Source: PLoS Comput Biol. 2017 Jul 26;13(7):e1005659. doi: 10.1371/journal.pcbi.1005659 (PMC5549999; doi:10.1371/journal.pcbi.1005659)
Supplement: S1 Source Code — (ZIP) [file pcbi.1005659.s002.zip › OpenMM-7.0.1-Source/docs/api-python/generated/simtk.openmm.openmm.AmoebaGeneralizedKirkwoodForce.html]

AmoebaGeneralizedKirkwoodForce — OpenMM 7.0.1.dev-5e86c4f documentation


# AmoebaGeneralizedKirkwoodForce¶

*class* `simtk.openmm.openmm.``AmoebaGeneralizedKirkwoodForce`(*\*args*)¶
:   This class implements an implicit solvation force using the generalized Kirkwood/Grycuk model.

    To use this class, create an AmoebaGeneralizedKirkwoodForce object, then call addParticle() once for each particle in the System to define its parameters. The number of particles for which you define parameters must be equal to the number of particles in the System, or else an exception will be thrown when you try to create a Context. After a particle has been added, you can modify its force field parameters by calling setParticleParameters(). This will have no effect on Contexts that already exist unless you call updateParametersInContext().

    `__init__`(*self*) → AmoebaGeneralizedKirkwoodForce¶
    :   \_\_init\_\_(self, other) -> AmoebaGeneralizedKirkwoodForce

        This class implements an implicit solvation force using the generalized Kirkwood/Grycuk model.

        To use this class, create an AmoebaGeneralizedKirkwoodForce object, then call addParticle() once for each particle in the System to define its parameters. The number of particles for which you define parameters must be equal to the number of particles in the System, or else an exception will be thrown when you try to create a Context. After a particle has been added, you can modify its force field parameters by calling setParticleParameters(). This will have no effect on Contexts that already exist unless you call updateParametersInContext().

    Methods

    |  |  |
    | --- | --- |
    | `__init__`(...) | \_\_init\_\_(self, other) -> AmoebaGeneralizedKirkwoodForce |
    | `addParticle`((self, charge, radius, ...) | Add the parameters for a particle. |
    | `getForceGroup`((self) -> int) | Get the force group this Force belongs to. |
    | `getIncludeCavityTerm`((self) -> int) | Get the flag signaling whether the cavity term should be included |
    | `getNumParticles`((self) -> int) | Get the number of particles in the system. |
    | `getParticleParameters`(self, index) | Get the force field parameters for a particle. |
    | `getProbeRadius`((self) -> double) | Get the probe radius (nm) used in SASA contribution |
    | `getSoluteDielectric`((self) -> double) | Get the dielectric constant for the solute. |
    | `getSolventDielectric`((self) -> double) | Get the dielectric constant for the solvent. |
    | `getSurfaceAreaFactor`((self) -> double) | Get the surface area factor kJ/(nm\*nm) used in SASA contribution |
    | `setForceGroup`(self, group) | Set the force group this Force belongs to. |
    | `setIncludeCavityTerm`(self, includeCavityTerm) | Set the flag signaling whether the cavity term should be included |
    | `setParticleParameters`(self, index, charge, ...) | Set the force field parameters for a particle. |
    | `setProbeRadius`(self, probeRadius) | Set the probe radius (nm) used in SASA contribution |
    | `setSoluteDielectric`(self, dielectric) | Set the dielectric constant for the solute. |
    | `setSolventDielectric`(self, dielectric) | Set the dielectric constant for the solvent. |
    | `setSurfaceAreaFactor`(self, surfaceAreaFactor) | Set the surface area factor kJ/(nm\*nm) used in SASA contribution |
    | `updateParametersInContext`(self, context) | Update the per-particle parameters in a Context to match those stored in this Force object. |
    | `usesPeriodicBoundaryConditions`((self) -> bool) | Returns whether or not this force makes use of periodic boundary conditions. |

    `getNumParticles`(*self*) → int¶
    :   Get the number of particles in the system.

    `addParticle`(*self*, *charge*, *radius*, *scalingFactor*) → int¶
    :   Add the parameters for a particle. This should be called once for each particle in the System. When it is called for the i’th time, it specifies the parameters for the i’th particle.

        |  |  |
        | --- | --- |
        | Parameters: | - **charge** (*double*) – the charge of the particle, measured in units of the proton charge - **radius** (*double*) – the atomic radius of the particle, measured in nm - **scalingFactor** (*double*) – the scaling factor for the particle |
        | Returns: | the index of the particle that was added |
        | Return type: | int |

    `getParticleParameters`(*self*, *index*)¶
    :   Get the force field parameters for a particle.

        |  |  |
        | --- | --- |
        | Parameters: | **index** (*int*) – the index of the particle for which to get parameters |
        | Returns: | - **charge** (*double*) –   the charge of the particle, measured in units of the proton charge - **radius** (*double*) –   the atomic radius of the particle, measured in nm - **scalingFactor** (*double*) –   the scaling factor for the particle |

    `setParticleParameters`(*self*, *index*, *charge*, *radius*, *scalingFactor*)¶
    :   Set the force field parameters for a particle.

        |  |  |
        | --- | --- |
        | Parameters: | - **index** (*int*) – the index of the particle for which to set parameters - **charge** (*double*) – the charge of the particle, measured in units of the proton charge - **radius** (*double*) – the atomic radius of the particle, measured in nm - **scalingFactor** (*double*) – the scaling factor for the particle |

    `getSolventDielectric`(*self*) → double¶
    :   Get the dielectric constant for the solvent.

    `setSolventDielectric`(*self*, *dielectric*)¶
    :   Set the dielectric constant for the solvent.

    `getSoluteDielectric`(*self*) → double¶
    :   Get the dielectric constant for the solute.

    `setSoluteDielectric`(*self*, *dielectric*)¶
    :   Set the dielectric constant for the solute.

    `getIncludeCavityTerm`(*self*) → int¶
    :   Get the flag signaling whether the cavity term should be included

    `setIncludeCavityTerm`(*self*, *includeCavityTerm*)¶
    :   Set the flag signaling whether the cavity term should be included

    `getProbeRadius`(*self*) → double¶
    :   Get the probe radius (nm) used in SASA contribution

    `setProbeRadius`(*self*, *probeRadius*)¶
    :   Set the probe radius (nm) used in SASA contribution

    `getSurfaceAreaFactor`(*self*) → double¶
    :   Get the surface area factor kJ/(nm\*nm) used in SASA contribution

    `setSurfaceAreaFactor`(*self*, *surfaceAreaFactor*)¶
    :   Set the surface area factor kJ/(nm\*nm) used in SASA contribution

    `updateParametersInContext`(*self*, *context*)¶
    :   Update the per-particle parameters in a Context to match those stored in this Force object. This method provides an efficient method to update certain parameters in an existing Context without needing to reinitialize it. Simply call setParticleParameters() to modify this object’s parameters, then call updateParametersInContext() to copy them over to the Context.

        The only information this method updates is the values of per-particle parameters. All other aspects of the Force (the probe radius, the surface area factor, etc.) are unaffected and can only be changed by reinitializing the Context.

    `usesPeriodicBoundaryConditions`(*self*) → bool¶
    :   Returns whether or not this force makes use of periodic boundary conditions.

        |  |  |
        | --- | --- |
        | Returns: | true if nonbondedMethod uses PBC and false otherwise |
        | Return type: | bool |

    `__delattr__`¶
    :   x.\_\_delattr\_\_(‘name’) <==> del x.name

    `__format__`()¶
    :   default object formatter

    `__getattribute__`¶
    :   x.\_\_getattribute\_\_(‘name’) <==> x.name

    `__hash__`¶

    `__reduce__`()¶
    :   helper for pickle

    `__reduce_ex__`()¶
    :   helper for pickle

    `__sizeof__`() → int¶
    :   size of object in memory, in bytes

    `__str__`¶

    `getForceGroup`(*self*) → int¶
    :   Get the force group this Force belongs to.

    `setForceGroup`(*self*, *group*)¶
    :   Set the force group this Force belongs to.

        |  |  |
        | --- | --- |
        | Parameters: | **group** (*int*) – the group index. Legal values are between 0 and 31 (inclusive). |

High performance molecular simulation on GPUs


### Navigation

- Application Layer
- Library Layer
  - Core Objects
  - Forces
  - Integrators
  - Extras

©2015, Stanford University and the Authors.
|
Powered by Sphinx 1.3.1
& Alabaster 0.7.7
|
Page source
